# IL-3: key orchestrator of inflammation

**DOI:** 10.3389/fimmu.2024.1411047

**Published:** 2024-06-13

**Authors:** Malgorzata J. Podolska, Robert Grützmann, Christian Pilarsky, Alan Bénard

**Affiliations:** Department of Surgery, Universitätsklinikum Erlangen, Friedrich-Alexander Universität Erlangen-Nürnberg, Erlangen, Germany

**Keywords:** interleukin-3, CD123, inflammation, infection, cancer

## Abstract

Interleukin (IL)-3 has long been known for its hematopoietic properties. However, recent evidence has expanded our understanding of IL-3 function by identifying IL-3 as a critical orchestrator of inflammation in a wide array of diseases. Depending on the type of disease, the course of inflammation, the cell or the tissue involved, IL-3 promotes either pathologic inflammation or its resolution. Here, we describe the cell-specific functions of IL-3 and summarize its role in diseases. We discuss the current treatments targeting IL-3 or its receptor, and highlight the potential and the limitations of targeting IL-3 in clinics.

## Introduction

**Figure 1:**
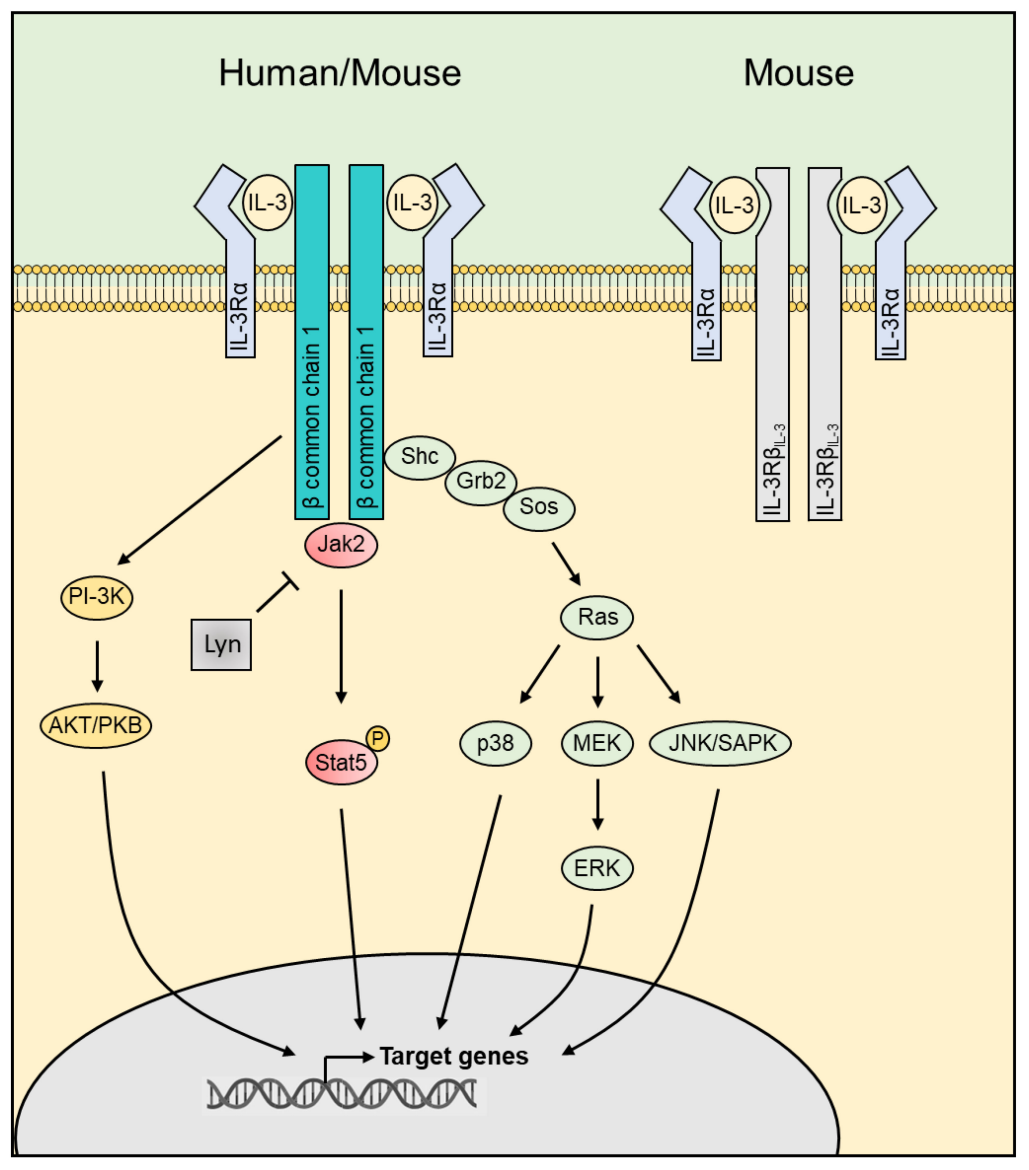
The IL-3 receptors and the downstream signaling. IL-3 exerts its function in humans through a heterodimeric receptor composed of the IL-3 receptor α-chain (CD123) and the common receptor β-chain (CD131). In addition to CD131, mice express another IL-3-specific β chain showing a strong homology at the amino acid level with CD131 but differing by its ability to bind IL-3. IL-3 binding leads to activation of downstream pathways including JAK2/STAT5, PI-3K/AKT and MAPK. JAK, Janus Kinase; STAT, signal transducer and activator of transcription; PI-3K, phosphoinositide 3-kinase; AKT, Protein kinase B; MAPK, Mitogen-Activated Protein Kinase.

**Figure 2:**
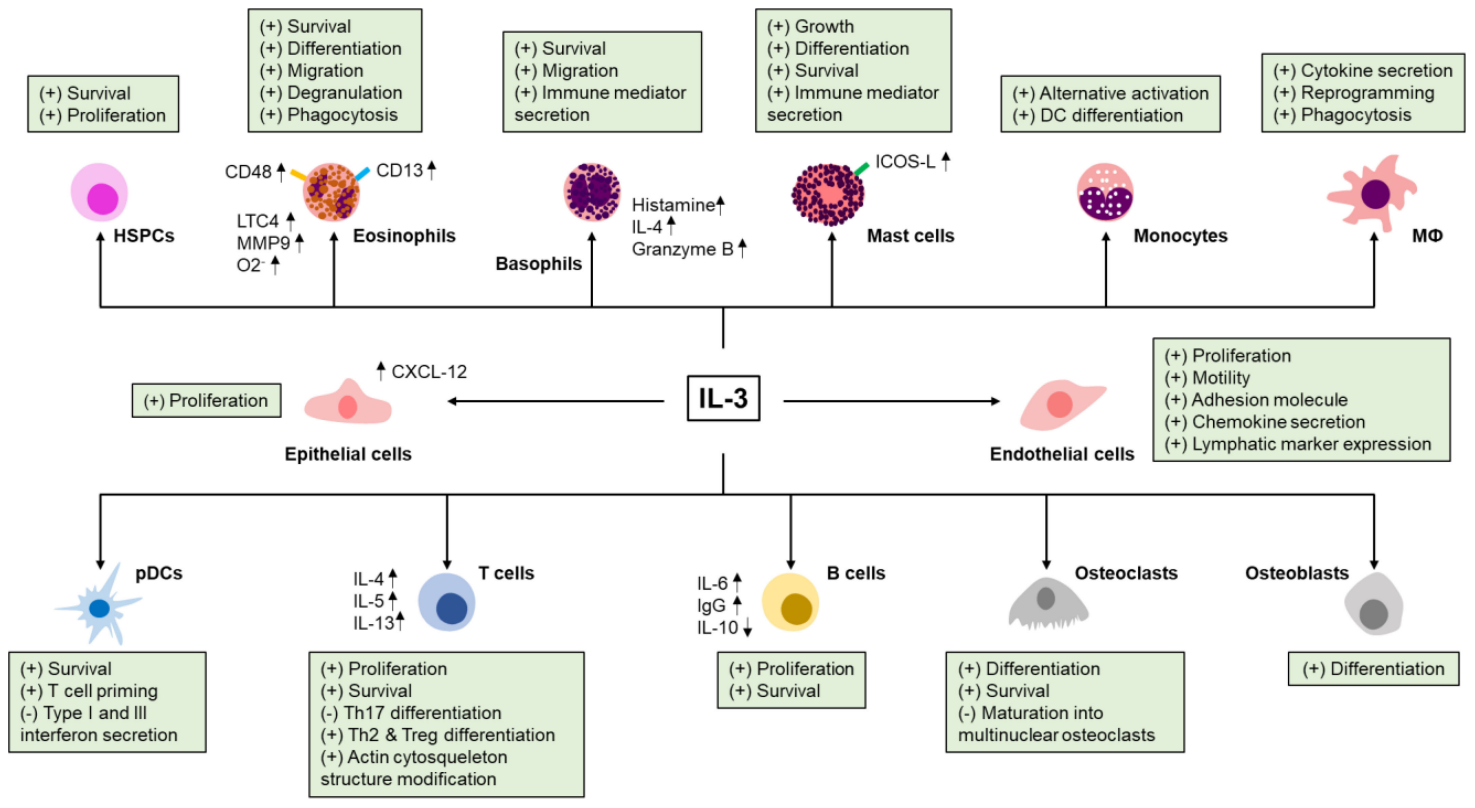
Effect of IL-3 on immune and non-immune cells. IL-3 modulates the survival, the growth, the differentiation, the migration, the phagocytosis and the secretion of immune mediators of many hematopoietic (hematopoietic stem and progenitor cells (HSPCs), eosinophils, basophils, mast cells, monocytes, macrophages, plasmacytoid dendritic cells (pDCs), T and B cells) and non-hematopoietic cells (endothelial cells, epithelial cells, osteoblasts and osteoclasts). CD, cluster of differentiation; LTC4, leukotriene C4; MMP9, matrix metallopeptidase 9; O2-, superoxide anion; IL-4, interleukin-4; ICOS-L, inducible costimulator-ligand; M⦶, macrophages; CXCL-12, C-X-C motif chemokine 12; Th, T helper; Treg, regulatory T cells; IgG, immunoglobulin G.

Interleukin (IL)-3, a cytokine belonging to the β common chain family of cytokines with IL-5 and granulocyte-macrophage colony-stimulating factor (GM-CSF), is mainly produced by immune cells, but also by some non-immune cells, such as astrocytes or cells harboring a mesenchymal stem cell (MSC) phenotype (1, 2). IL-3 exerts its function through a heterodimeric receptor composed of the IL-3 receptor α-chain (CD123) and the common receptor β-chain (CD131), CD123 providing the specificity for IL-3, while CD131 is essential for signaling and assembly (3). Given the low affinity of IL-3 for CD123, heterodimerization with CD131 is crucial as it creates a high-affinity receptor complex (3). In addition to CD131, mice express another IL-3-specific β chain, this chain differing from CD131 in its ability to bind murine IL-3 directly (4), although the presence of CD123 is required for signaling (5) (**Figure 1**). Thus, the expression pattern of CD123 defines the target cell profiles and function of IL-3. CD123 is mainly expressed on hematopoietic cells, including hematopoietic stem and progenitor cells (HSPCs), basophils, eosinophils, mast cells, non-classical monocytes, macrophages, human plasmacytoid dendritic cells (pDCs) and activated T and B cells. CD123 expression is not limited to the hematopoietic compartment, but it extends to non-hematopoietic cells, such as endothelial and epithelial cells or osteoblasts and osteoclasts (**Figure 2**).

Blood levels of IL-3 are usually low to undetectable in healthy donors, although some have high IL-3 levels (6, 7). Likewise, IL-3 is hardly detected in the healthy or non-inflammatory conditions of many tissues but is produced at the site of inflammation, revealing IL-3 as an important regulator of the immune response. Indeed, IL-3 supports the survival, the proliferation, the differentiation, the polarization or the recruitment of immune and non-immune cells in infection, inflammatory diseases and cancer (**Table 1**).

**Table 1:** Role of IL-3 in diseases.

Disease	Effect	Mechanism	Reference
Autoimmune myocarditis	Detrimental	Amplify CCR2^+^ Ly-6C^high^ monocyte chemotaxis.	(8)
Allograft heart transplantation	Detrimental	Induce fibrosis by enhancing basophil mediator release.	(9)
Atherosclerosis	Detrimental	Support VSMC proliferation and migration. Stimulate splenic extramedullary hematopoiesis.	(10–12)
SLE	Detrimental	Potentiate IFNα, IFNλ1 and IFNλ3 expression.	(7, 13)
Multiple sclerosis/EAE	Detrimental	Amplify CNS immune cell infiltration.	(14, 15)
Hematologic cancers	Detrimental	Stimulate tumor cell proliferation and survival. Increase bone loss (MM).	(16–18)
Alzheimer disease	Protective	Reprogram microglia into a protective acute immune response program.	(1)
Arthritis	Protective	Prevent cartilage and bone loss in the joints. Reduce matrix metalloproteinase expression. Induce regulatory T cell development Prevent Th17 development.	(19–24)
Diabetes	Protective	Induce immature T cell development with strong immunoregulatory function.	(25, 26)
Viral infections	Protective	Enhance pDC recruitment into lung parenchyma. Enhance pDC-induced T cell priming. Induce IFNλ expression.	(27–29)
Asthma	Detrimental Protective	Enhance histamine and Th2 cytokine secretion by basophils. Reduce innate lymphoid cell type 2 number. Increase regulatory T cell number.	(30–32)
Parasitic infections	Detrimental Protective	Alter macrophage antimicrobial activity. Promote basophil and mast cell recruitment and function.	(33–39)
Bacterial infections	Detrimental Protective	Potentiate LPS and *Pseudomonas aeruginosa* inflammation. Stimulate splenic extramedullary hematopoiesis N.D. (*S.Typhimurium*).	(40–42)
Inflammatory bowel diseases	Detrimental Protective	Stimulate splenic extramedullary hematopoiesis. Promote early recruitment of splenic neutrophils into the colon. Prevent regulatory T cell egress from the colon. Stimulate basophil proliferation.	(2, 43, 44)
Solid cancers	Detrimental Protective	Stimulate basophil-produced IL-4 in draining lymph node (PDAC). Promote epithelial-to-endothelial and epithelial-to-mesenchymal transition (breast cancer). Promote lung metastasis (breast cancer). Increase PD-L1 expression (breast cancer). Stimulate angiogenesis (breast cancer). Enhance tumor-specific cytotoxic CD8^+^ T cell generation (lung carcinoma).	(45–47)

CCR2, C-C chemokine receptor type 2; Ly6C, lymphocyte antigen 6 complex; VSMC, vascular smooth muscle cell; EAE, experimental autoimmune encephalomyelitis; CNS, central nervous system; Th, T helper; SLE, systemic lupus erythematosus; IFN, interferon; pDC, plasmacytoid dendritic cells; LPS, lipopolysaccharide; MM, multiple myeloma; PDAC, pancreatic ductal adenocarcinoma; PD-L, programmed death-ligand 1; CD, cluster of differentiation.

Here, we review the function of IL-3 in health and disease. We describe the cell-specific effects of IL-3 and summarize its role in host defense, autoimmunity, and cancer. Likewise, we discuss the current treatments targeting IL-3 or its receptor and highlight the potential and the limitations of targeting IL-3 in clinics.

## Cellular targets of IL-3

### HSPCs

HSPCs give rise to all types of blood cells and play a pivotal role in building the immune system. As suggested by its former name (multi-colony stimulating factor), IL-3 stimulates the growth and differentiation of HSPCs from bone marrow cultures and in naive or sublethally irradiated mice into a large range of cell lineages, including basophils, neutrophils, eosinophils, macrophages, erythroid cells, megakaryocytes, and dendritic cells (DCs) (48, 49). Despite the abovementioned ability of IL-3 to stimulate hematopoiesis, mice deficient for *Il-3* exhibit normal hematopoiesis under physiological conditions (33) indicating that IL-3 is not essential for the development of all blood cell lineages at steady state. However, IL-3 was described to play a key role in splenic extramedullary hematopoiesis during inflammatory diseases (2, 10, 40), which is consistent with the ability of recombinant IL-3 to increase hematopoiesis in the spleen and the liver of naive mice but not in the bone marrow (48, 49). Likewise, IL-3 increases the number of tissue mast cells and enhances basophil production during parasite infection, while it is dispensable for their generation under physiological conditions (33), thus making IL-3 an orchestrator of emergency hematopoiesis.

### Eosinophils

Eosinophils are polymorphonuclear cells involved in the protection against multicellular parasites through the release of a variety of granular mediators and the production of toxic reactive oxygen species (50). IL-3 induces eosinophil survival (51) and is associated with early and end stages of eosinophilic differentiation and maturation (52). In addition, IL-3 increases human eosinophil adhesion, chemotaxis and migration (53) and stimulates cytotoxicity against antibody-sensitized helminths, superoxide anion production and phagocytosis of opsonized yeast particles (54).

### Basophils

Basophils, one of the main effector cells during allergic diseases, are described to express both IL-3 (55) and its receptor (56). As for eosinophils, IL-3 plays a key role in basophil survival, migration and activation. Indeed, IL-3 protects basophil from apoptosis *in vitro* in a Pim1-dependent manner (57), increases basophil number in the blood during parasite infection (34), and enables basophil extravasation during allergic contact dermatitis (58). Moreover, IL-3, alone or in a combination with other molecules, such as anti-FcϵRI mAb, complement component 5a, N-Formylmethionine-leucyl-phenylalanine (fMLP), CCL2 or eotaxin, enhances the expression of various immune mediators important for the function of basophils, such as histamine (59) or granzyme B (60).

### Mast cells

In mice, IL-3 promotes mast cell growth and differentiation (61) and enhances mediator release (62). However, CD123 was not detected on human mast cells purified from tonsils, lungs, uterus and skin (63) and no IL-3-binding sites were detected on enriched human lung mast cells (64) suggesting a limited impact of IL-3 on human mature mast cells *in vivo*.

### Myeloid cells and pDCs

In steady-state, murine monocytes and human classical monocytes do not express the receptor for IL-3. Only human intermediate and non-classical monocytes express it (65). Although IL-3 alone does not seem to modulate the function of CD14^+^ monocytes, *in vitro* experiments show that IL-3 synergizes with IL-4 to enhance CCL17 expression in CD14^+^ monocytes, a marker of alternative activation, IL-4 increasing the responsiveness to IL-3 by inducing CD123 expression (66). Likewise, IL-3 enhances the production of TNFα by CD14^+^ monocytes upon lipopolysaccharide (LPS) stimulation by modulating TNFα posttranscriptional levels (67). In combination with IFNβ or IL-4, IL-3 drives the differentiation of CD14^+^ monocytes into DCs exhibiting pro- or anti-inflammatory properties, respectively (68, 69), and IL-3 induces the expression of the lectins mannose receptor, Dectin-1, and DC-SIGN during monocyte-derived macrophage differentiation allowing increased phagocytosis (70). Besides this, IL-3 modulates the expression of IL-1 and the chemokines CCL2, CCL3, CCL7, CCL12 in macrophages (8, 71, 72), increases peritoneal macrophage phagocytic activity (48), and elicits transcriptional, morphological, and functional programming of microglia (1, 14).

In addition to the monocyte-macrophage compartment, it was described that human pDCs express high levels of CD123, whereas murine pDCs do not express it (73). In humans, IL-3 promotes the survival of pDCs (74) as well as their differentiation in a sub-population of DCs characterized by a low ability to produce type I and III interferons but with a high capacity to prime T cells (27, 28). Interestingly, recent studies have revealed that CD123 is not only expressed on human pDCs but also on AXL^+^ DCs, dendritic cell precursors that look similar to conventional DC2s in terms of basic function and morphology (75).

### T and B cells

T cells are a major source of IL-3 during inflammation (76) and IL-3-producing T cells are involved in many inflammatory and infectious diseases. In humans, CD123 is expressed on activated CD4^+^ and CD8^+^ T cells (77). Exposure to IL-3 enhances T cell proliferation and survival (77), inhibits Th17 differentiation (19) and enhances Th2 differentiation as well as IL-4, IL-5, and IL-13 expressions (78). Moreover, IL-3 promotes the differentiation of naive CD4^+^ T cells into regulatory T cells, and modulates regulatory T cell migration by modifying their actin cytoskeleton structure (43). Like T cells, activated human B cells express CD123 and IL-3 supports their proliferation and survival (77). In addition, IL-3 enhances IgG and IL-6 secretions but reduces IL-10 expression (77, 79). Recently, innate response activator (IRA) B cells, a subset of B-1a B cells residing in serosal sites, have been described as a source of IL-3 in infectious and inflammatory diseases (2, 40). After activation, IL-3-producing IRA B cells accumulate in the spleen, where they fuel the immune response by promoting extramedullary hematopoiesis (2, 40).

### Endothelial and epithelial cells

In addition to hematopoietic cells, CD123 is expressed by endothelial and epithelial cells (2, 29). IL-3 induces intestinal epithelial cell proliferation (80) and stimulates CXCL12 expression in lung epithelial cells (29), while it promotes endothelial cell proliferation and motility *in vitro* (81, 82) as well as new vessel formation and tumor angiogenesis *in vivo* (82). Moreover, IL-3 induces the expression of adhesion molecules and chemokines in endothelial cells, thereby promoting immune cell rolling, adhesion, and transmigration (81).

### Osteoblasts and osteoclasts

IL-3 plays an important role in bones by directly targeting both osteoblasts and osteoclasts. IL-3 promotes the differentiation of human mesenchymal stem cells into osteoblasts (83) and stimulates the formation and the survival of mononuclear osteoclasts (84). However, IL-3 inhibits the formation of mature multinucleated osteoclasts by diverting osteoclast precursors into macrophage or dendritic cell lineages, inhibiting NF-kB nuclear translocation induced by RANKL, and downregulating the expression of c-Fms (20, 21).

## IL-3 in diseases

### Cardiovascular diseases

Cardiovascular diseases are a group of disorders of the heart and blood vessels that are a leading cause of mortality worldwide (85). Although IL-3 is hardly detected in the heart in steady-state, its expression increased significantly during autoimmune myocarditis (8) and allograft heart transplantation (9). IL-3 is also expressed in early and advanced atherosclerotic plaques in humans (11) and plasma IL-3 levels predict for symptomatic restenosis (86). In experimental autoimmune myocarditis, IL-3 amplifies cardiac inflammation by promoting CCR2^+^ Ly-6C^high^ monocyte accumulation in the heart via the stimulation of tissue macrophages. Recruited monocytes give rise to APCs that enhance local T cell proliferation and T cell-derived cytokine production, including IL-3, thus amplifying local inflammation (8). In addition, deletion or inhibition of IL-3 reduced the development of myocardial fibrosis and protects from chronic rejection of heart transplants by reducing the ability of infiltrating basophil to secrete IL-4 and IL-6 (9). In atherosclerosis, IL-3 sustains both the proliferation and the migration of vascular smooth muscle cells (11), a major source of plaque cells and extracellular matrix at all stages of atherosclerosis (87). Interestingly, IL-3 secreted in the spleen of *Apoe^-/-^* mice promotes HSPC expansion and differentiation into Ly6C^high^ monocytes. Monocytes born in such extramedullary niches intravasate, circulate, and accumulate abundantly in atherosclerotic lesions where they secrete inflammatory cytokines, reactive oxygen species and proteases, and eventually become foam cells (10), thus promoting atheroma macrophage burden (12). Therefore, IL-3 seems to modulate inflammation in cardiac and vascular tissue by exerting its activity directly at the inflammatory site and indirectly in periphery.

### Autoimmune diseases

#### Arthritis

In a model of inflammatory arthritis, IL-3 inhibits TNFα-induced bone resorption and prevents cartilage and bone loss in the joints (22). In osteoarthritis, IL-3 protects from cartilage degeneration and bone damage by reducing the expression of matrix metalloproteinases (MMPs) (23). IL-3 also reduces the severity of collagen-induced arthritis by modulating the development of Foxp3 regulatory T-cells (24) and by preventing the development of Th17 cells (19). Thus, it seems that IL-3 protects from joint inflammation by modulating the immune response in arthritis in addition to its role on osteoblast and osteoclast differentiation.

#### Diabetes

Diabetes is a chronic metabolic disease characterized by high levels of glucose in the blood. There are two main types of diabetes: type 1 diabetes (T1D) and type 2 diabetes (T2D). T1D develops when the immune system attacks and destroys pancreatic beta cells resulting in an inability of patients to produce insulin (88). T2D is characterized by a reduced production of insulin or a resistance to it, and is associated with obesity or high body fat percentage in the abdominal region (89). While its role during T2D is still unknown, IL-3 is thought to be protective during T1D. Indeed, IL-3 stimulates the development of immature T cells with strong immunoregulatory function in the bone marrow of non-obese diabetic (NOD) mice, which significantly delays the apparition of the first symptoms and reduces the overall incidence of the disease (25). In addition, deficiency in *Il-3* and *Csf2* results in insulitis, insulin-producing β cell destruction, and abnormal glucose tolerance (26).

#### Central nervous system inflammation

In the central nervous system (CNS), IL-3 is mainly expressed by neurons and a subset of astrocytes (1, 90). In patients with Alzheimer disease (AD), low plasma IL-3 levels are associated with AD risk (91), whereas IL-3 levels in frontal cortex tissue are unaltered by AD pathology (1). Only the expression of CD123 is increased in frontal cortex tissue and correlates with disease duration and β-amyloid (Aβ) levels (1). This increase of CD123 is specific to microglia, macrophages of the CNS, and is age-dependent (1). The stimulation of CD123^+^ microglia by IL-3 induces their reprogramming into an acute immune response program that improves their ability to clear Aβ and tau aggregates, thus limiting AD (1). By contrast, IL-3 seems to be detrimental in multiple sclerosis (MS). For instance, IL-3 administration to mice with experimental autoimmune encephalitis (EAE) worsens the disease (15), whereas mice deficient for *Il-3* are protected from developing EAE (14). In MS, IL-3 is produced by astrocytes and infiltrating CD44^hi^CD4^+^ T cells, while CD123 is mainly expressed by microglia and recruited myeloid cells in the spinal cord (14). As observed in AD, the local production of IL-3 reprograms IL-3Rα^+^ myeloid cells of the CNS. However, this reprogramming amplifies CNS immune cell infiltration increasing MS severity. Thus, this discrepancy between AD and MS reveals a dual role of IL-3 during CNS inflammation, the same mechanism (reprogramming of IL-3R^+^ myeloid cells) leading to two different outcomes (beneficial in AD and detrimental in MS).

#### Systemic lupus erythematosus

In MLR/lpr mice, a mouse model of systemic lupus erythematosus, plasma IL-3 levels increase concomitantly with disease progression. Administration of IL-3 aggravates lupus nephritis in MLR/lpr mice, whereas injection of antibodies against IL-3 reduces the severity of the disease (92). In patients with SLE, pDCs are continuously activated by circulating immune complexes resulting in an aberrant production of type I IFNs, which contributes to autoreactive T cells stimulation and autoantibody-secreting plasma cell generation, thus highlighting IFN and pDCs as critical contributors to the disease. Interestingly, serum IL-3 levels correlate with serum IFNα and IFNλ levels in patients with SLE (7) and IL-3 potentiates the secretion of IFNα by pDCs upon immune complexes stimulation (13). Targeting CD123^+^ cells or IL-3 signaling could therefore constitute a future therapeutic target in SLE.

### Asthma

Studies investigating the contribution of IL-3 in asthma pathogenesis, a chronic inflammatory disorder associated with airway hyper-responsiveness, show inconsistent results, both on IL-3 expression levels and function. Indeed, IL-3 levels were found either increased in sputum (93) and BALF (94) of asthmatic patients, reduced in nasopharyngeal fluid of asthmatic children (30), or similar in bronchial biopsies between asthmatic and non-asthmatic patients (95). Likewise, a report showed that mice deficient for *Il-3* exhibit increased pulmonary inflammation during asthma, characterized by local eosinophil infiltration and increased secretion of IgE, IL-5, and IL-13 (31). On the contrary, another study reported that *Il-3* ^-/-^ mice show similar number of basophils and eosinophils in BALF as well as similar serum IgE levels when compared to control mice (32). In human, it was shown that (i) sputum IL-3 secretion correlates with levels of eosinophil granule proteins and decreased lung function (93); (ii) IL-3^+^ BALF cells are associated with asthma severity (96) and; (iii) serum IL-3 levels are higher in patients with uncontrolled chronic asthma (97). By contrast, other studies revealed that IL-3 produced by PBMCs is associated with amelioration of asthma in pre-school children (31) and that IL-3 expression in lung tissue is not correlated with eosinophil and metachromatic cell number, airway responsiveness, or the severity of the late asthmatic response (95). Mechanistically, IL-3 was described to protect against asthma by reducing the number of innate lymphoid cell type 2 in lungs and by increasing regulatory T cell number in the airways (30, 31). On the other hand, IL-3 was reported to be detrimental in asthma by enhancing the secretion of histamine and Th2 cytokines by basophils (32). This contradictory effect in mice might result from the different times of injection, application routes, and doses of IL-3, while the differences observed in humans might be explained by the age of the patients (children vs adults) or the stage of the disease.

### Infectious diseases

#### Parasitic infections

Protozoa and helminths are the two main parasites infecting humans. Interestingly, polymorphism in the human *IL-3* gene was described to be associated with the pathophysiology of malaria (98) and mice lacking IL-3 are more resistant to *Plasmodium berghei* NK65-induced cerebral malaria (35). Moreover, mice susceptible to *Leishmania major* infections exhibit higher IL-3-secreting draining lymph node cell number than resistant mice and reduced disease score after anti-IL-3 antibody treatment (36). Thus, these results suggest that IL-3 impairs protective immunity during protozoan infections. The mechanisms associated with IL-3-dependent susceptibility to protozoan infections seem to involve macrophages as shown by (i) the ability of mast cell-produced IL-3 to alter the antimicrobial activity of macrophages during *Leishmania donovani* infections (37) and; (ii) the aggravation of *Leishmania major* infections after adoptive transfer of macrophages treated with IL-3 (36). Unlike protozoan infections, *Il-3*-deficient mice exhibited defective immunity against *Strongyloides venezuelensis* (33) and treatment with recombinant IL-3 protects C57BL/6 mice against *Strongyloides ratti* but not against *Nippostrongylus brasiliensis* (38), indicating that IL-3 protects against some helminth infections. The anti-helminthic effect of IL-3 is exerted by its ability to increase the number of circulating basophils and tissue mast cells, to promote the recruitment of basophils into the lymph node, and to stimulate the secretion of IL-4 by basophils (33, 34, 39).

#### Viral infections

IL-3 was described as a predictive marker for clinical outcome and disease severity during SARS-CoV-2^+^ infections (29), IL-3 being mainly produced by CD4^+^ T cells (29) and induced by the viral ORF7a protein (99). In a mouse model of pulmonary HSV-1 infection, IL-3 protects against viral pneumonia by promoting the recruitment of pDC into the lung parenchyma in a CXCL12-dependent manner (29). In humans, IL-3 enhances T cell priming by pDCs but have no effect on type I or type III IFN production by pDCs upon viral activation (27, 28). Moreover, plasma IL-3 and IFNλ levels correlate in patients with SARS-CoV-2^+^ infections and in septic patients with pulmonary viral infections suggesting that IL-3 might also induce IFNλ expression during viral airway infections in a pDC-independent manner (28, 29). Thus, IL-3 seems to play a critical role in safeguarding environments frequently exposed to viruses.

#### Bacterial infections

The role of IL-3 during bacterial infections remains poorly described. Studies using LPS, a membrane component of Gram-negative bacteria, show that IL-3 enhances pro-inflammatory cellular response to LPS *in vitro* (41). *In vivo*, IL-3 augments LPS-induced murine lung injury by enhancing the recruitment of neutrophils and lymphocytes as well as the secretion of pro-inflammatory cytokines in BALF (42). Also, IL-3 potentiates inflammation during cecal ligature and puncture-induced sepsis by promoting the myelopoiesis of monocytes and neutrophils, which fuels the cytokine storm and leads to increased mortality (40) but protects against *S. Typhimurium* infections (2).

### Inflammatory bowel diseases

Inflammatory bowel diseases (IBDs) are inflammatory disorders of the intestinal tract characterized by in an aberrant mucosal immune response driven by microbial factors of the commensal gut microbiota (100). Whereas the role of GM-CSF during colitis is clearly established, the role of IL-3 was until recently unknown. IL-3 and CD123 expressions are upregulated in inflamed tissue of patients with IBDs and correlate with the levels of inflammation (2, 43). In the colon, IL-3 is produced by T cells and cells harboring a mesenchymal stem cell phenotype, whereas CD123 is expressed by epithelial and infiltrating immune cells (2, 43). The use of different models of colitis has revealed that IL-3 has a dual role during acute colitis, which seems to depend on the intensity of the inflammation. On the one hand, IL-3 protects by (i) promoting the early recruitment of splenic neutrophils with high microbicidal capacity in the colon, which requires CCL5^+^ PD-1^hi^ LAG-3^hi^ T cells, STAT5 signaling, and CCL20 (2); (ii) preventing the egress of regulatory T cells from the colon through the modification of their actin cytoskeleton structure (43) and; (iii) stimulating the proliferation of basophils, which protects from acute inflammation by reducing the expression of IFNγ, IL-2, and TNFα in T cells (44). On the other hand, it was reported that IL-3 increases colitis severity during the acute phase of colitis by amplifying colonic inflammation (2). This phenomenon might result from the ability of IL-3 to promote extramedullary hematopoiesis in the spleen during colitis (2), which is important at the onset of the disease to sustain splenic neutrophil emigration into the colon, but might become detrimental during acute colitis by fueling the inflammatory immune response.

### Cancers

#### Hematologic cancers

Hematologic cancers are characterized by an accumulation of abnormal hematopoietic cells in the bone marrow, blood or lymph nodes (101). As observed during normal hematopoiesis, IL-3 acts as a growth factor in many hematologic neoplasms. While expressed at low levels or absent on normal hematopoietic stem cells, CD123 is overexpressed in a variety of hematologic cancers, such as acute myeloid leukemia (AML), blastic plasmacytoid dendritic cell neoplasm (BPDCN), B-cell acute lymphoblastic leukemia, or Hodgkin lymphoma (3). In AML, high expression of CD123 on blasts or leukemic stem cells is associated with increased blast proliferation, resistance to apoptosis and worst prognosis (16). In addition, it enhances the response to low concentration of IL-3 and alters CXCR4/CXCL12 interaction in the BM (17). Moreover, IL-3 is significantly increased in serum of children with AML as well as in the bone marrow plasma of patients with multiple myeloma (MM), while no difference was observed in children with acute lymphoblastic leukemia (ALL) (18).

#### Solid cancers

Unlike hematologic cancers, the role of IL-3 in solid tumors is little described. In patients with pancreatic ductal adenocarcinomas (PDAC), serum IL-3 levels are significantly lower than in healthy donors and do not correlate with the most common symptoms, the tumor location and the cancer stage (102). In PDAC-draining lymph nodes, IL-3, produced by CD4^+^ T cells, stimulates basophils to secrete IL-4, which is necessary for the stabilization of the Th2 phenotype (45), a signature associated with reduced survival. In triple negative breast cancer, CD123 expression correlates with nodal metastasis and reduced survival (46), and IL-3 promotes epithelial-to-endothelial and epithelial-to-mesenchymal transition, lung metastasis, and increased programmed-cell death ligand-1 (PD-L1) expression on tumor and mononuclear immune cells (46). Finally, it was reported that serum IL-3 levels may act as a tumor marker for colorectal cancer (103) and that overexpression of IL-3 in a mouse lung carcinoma cell line inhibits tumor growth *in vivo* by enhancing the generation of tumor-specific cytotoxic CD8^+^ T cells (47).

## IL-3 and CD123 in clinics

**Figure 3:**
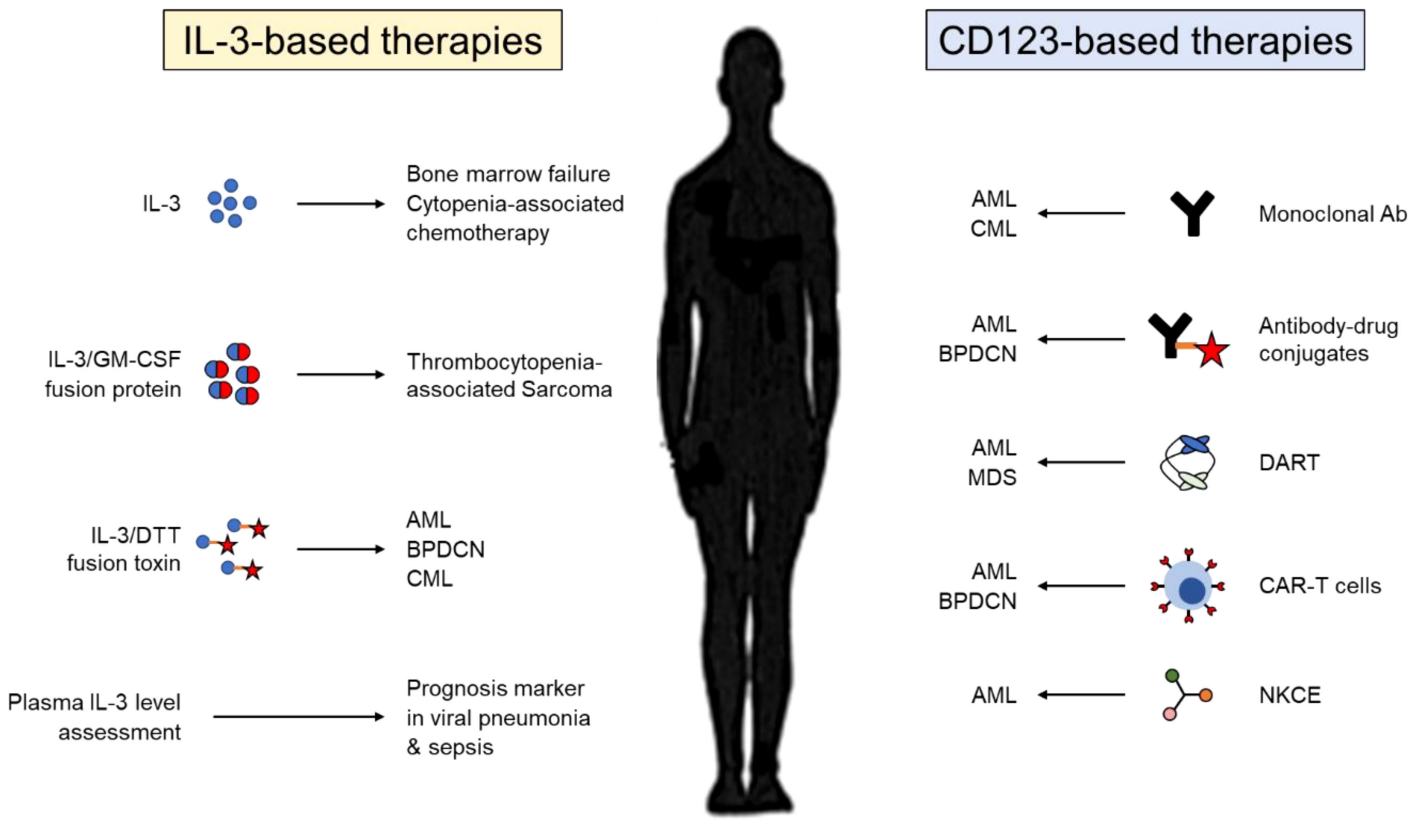
Therapeutic targeting of IL-3 and its receptor CD123. Therapies used in pre-clinical or clinical studies targeting either IL-3 (left) or CD123 (right). IL-3, interleukin-3; GM-CSF; granulocyte-macrophage colony-stimulating factor; DTT, diphtheria toxin; AML, acute myeloid leukemia; BPDCN, blastic plasmacytoid dendritic cell neoplasm; CML, chronic myelogenous leukemia; Ab, antibody; MDS, myelodysplastic syndrome; DART, dual affinity retargeting molecule; CAR-T cells, chimeric antigen receptor T cells; NKCE, natural killer cell engager.

Given the ability of IL-3 to stimulate hematopoiesis, multiple clinical trials have evaluated IL-3 as a treatment for patients with cytopenia (**Figure 3**). Administration of IL-3 in healthy subjects and patients with bone marrow failure or myelodysplastic syndrome results in increased platelet, reticulocyte and leukocyte numbers with only mild side effects (104). Likewise, IL-3 alone or followed by G-CSF reduces the incidence and the severity of neutropenia and/or thrombocytopenia associated with chemotherapy in patients with solid tumors (105). Thus, IL-3 alone effectively abrogates cytopenia, but does not appear to have a real benefit after chemotherapy in comparison with GM-CSF or G-CSF. As well, IL-3 has limited effect in Human Immunodeficiency Virus (HIV)-infected patients with cytopenia (106). A fusion protein combining the active domain of IL-3 and GM-CSF was also assessed in clinical trials. This protein was described to be more efficient than GM-CSF in preventing cumulative thrombocytopenia associated with chemotherapy in patients with sarcoma but not in patients with advanced breast cancer (107, 108).

As previously described, myeloid and lymphoid leukemic stem cells express high levels of CD123 in comparison with normal hematopoietic stem cells pointing out CD123 as a potential target for therapy against leukemia (**Figure 3**). Early treatments have used either monoclonal antibodies against CD123 or antibody-drug conjugates against CD123. While preclinical studies showed promising results (109, 110), many clinical trials revealed either insufficient antileukemic activity or high toxicity (111). By contrast, therapies using a recombinant toxin combining the catalytic and translocation domains of diphtheria toxin and human IL-3 show promising efficacity for the treatment of leukemias (112). Preclinical studies have also demonstrated that redirecting specifically polyclonal T cells or natural killer (NK) cells against CD123^+^ tumor cells, using either chimeric antigen receptor (CAR) T cells, bispecific antibodies or NK cell engager molecules, might be promising therapeutic option in the future (113–115). For that, it will be important to maximize affinity to CD123 in order to have an acceptable therapeutic effect between tumor eradication and toxicity.

## Concluding remarks

Although the discovery of IL-3 dates back several decades, our knowledge on its function in immunity was quite limited, since most of the early studies focused on the hematopoietic properties of IL-3. However, recent studies have expanded our understanding of IL-3 function by identifying IL-3 as a critical orchestrator of inflammation in autoimmune diseases, cancer, and infection. Depending on the type of disease, the cell or the tissue involved, IL-3 can be either detrimental or beneficial. In addition to its role as a growth factor in cancers, IL-3 exerts its pathologic effect through the recruitment of monocytes into the inflammatory site (MS, experimental autoimmune myocarditis), the induction of inflammatory cytokines (asthma, SLE), or the stimulation of extramedullary hematopoiesis (sepsis, acute colitis). By contrast, IL-3 protects through the modulation of regulatory T cell development and migration (colitis, arthritis), the functional reprogramming of immune cells (AD, viral infection) and the early recruitment of splenic neutrophils or pDCs (early colitis, viral pneumonia). Moreover, through its ability to induce a Th2 response, IL-3 protects during helminth infection but is detrimental during asthma, protozoan infections and pancreatic cancers. Assessment of plasma IL-3 levels may therefore serve as a prognostic marker to identify patients at risk of developing severe disease. Since there are new technical possibilities to determine the plasma IL-3 value in a fast, affordable and at point-of-care manner (6), clinicians could adapt the therapeutic strategy for patients with the worst outcome. This is all the more important that genetic polymorphism of IL-3 or its receptor, which modulates IL-3 expression and signaling, has been identified as a potential factor in the pathophysiology of many diseases, such as Graves’ disease (116), schizophrenia (117, 118), atopy and asthma (119), acute kidney rejection (120) and rhinoconjunctivitis (121).

Given the impact of IL-3 in many inflammatory and infectious diseases, it would be interesting to assess if the therapies used in clinic for treating bone marrow failure or hematologic disorders may have a significant effect on the outcome of these diseases. It would be also of a potential interest to generate therapeutics with novel properties, such as dominant negative cytokines or drugs preventing either the heterodimerization of the IL-3 receptor or IL-3 expression itself. However, targeting IL-3 as a novel therapeutic approach may have ambivalent effects in certain diseases. Indeed, recent studies have highlighted that IL-3 may have a dual role depending on the course of the disease. In sepsis, IL-3 is detrimental during the acute phase by fueling the innate immune response but protects against pulmonary viral reactivation by improving antiviral defense mechanism. In colitis, IL-3 has a beneficial role at the onset of the disease by promoting the recruitment of splenic neutrophils with high microbicidal capability into the colon, whereas it has a detrimental effect during severe colitis by amplifying intestinal inflammation. Thus, it will be essential to identify in depth the cellular and molecular mechanisms associated with these dual effects for developing appropriated therapies.

For many years, IL-3 has been in the shadow of GM-CSF, but only recently has it been shown to be as important as GM-CSF in the regulation of inflammation. Nonetheless, the role of IL-3 is still elusive in many diseases, especially in solid cancers, so further research is still needed.

## Author contributions

MP: Writing – original draft, Writing – review & editing. RG: Writing – review & editing. CP: Writing – review & editing. AB: Writing – original draft, Writing – review & editing.
